# Protection of dopaminergic neurons in hemiparkinsonian monkeys by flavouring ingredient glyceryl tribenzoate

**DOI:** 10.1515/nipt-2022-0005

**Published:** 2022-06-08

**Authors:** Suresh B. Rangasamy, Debashis Dutta, Susanta Mondal, Moumita Majumder, Sridevi Dasarathy, Goutam Chandra, Kalipada Pahan

**Affiliations:** Department of Neurological Sciences, Rush University Medical Center, Chicago, USA; Department of Neurological Sciences, Rush University Medical Center, Chicago, USA; Department of Neurological Sciences, Rush University Medical Center, Chicago, USA; Department of Neurological Sciences, Rush University Medical Center, Chicago, USA; Department of Neurological Sciences, Rush University Medical Center, Chicago, USA; Department of Neurological Sciences, Rush University Medical Center, Chicago, USA; Department of Neurological Sciences, Rush University Medical Center, 1735 West Harrison St Suite Cohn 310 IL 60612, Chicago, USA; and Division of Research and Development, Jesse Brown Veterans Affairs Medical Center, Chicago, USA

**Keywords:** glyceryl tribenzoate, inflammation, microglia, MPTP, Parkinson’s disease

## Abstract

Parkinson’s disease (PD) is the second most prevalent neurodegenerative disease and this study underlines the significance of a small molecule glyceryl tribenzoate (GTB), a FDA approved food additive, in preventing parkinsonian pathologies in MPTP-induced animal models. The study conducted in MPTP-induced mice demonstrated dose-dependent protection of nigral tyrosine hydroxylase (TH) and striatal dopamine level by GTB oral treatment and the optimum dose was found to be 50 mg/kg/d. In the next phase, the study was carried out in MPTP-injected hemiparkinsonian monkeys, which recapitulate better clinical parkinsonian syndromes. GTB inhibited MPTP-driven induction of glial inflammation, which was evidenced by reduced level of GTP-p21^Ras^ and phospho-p65 in SN of monkeys. It led to decreased expression of inflammatory markers such as IL-1*β* and iNOS. Simultaneously, GTB oral treatment protected nigral TH cells, striatal dopamine, and improved motor behaviour of hemiparkinsonian monkeys. Presence of sodium benzoate, a GTB metabolite and a FDA-approved drug for urea cycle disorders and glycine encephalopathy, in the brain suggests that the neuroprotective effect imparted by GTB might be mediated by sodium benzoate. Although the mechanism of action of GTB is poorly understood, the study sheds light on the therapeutic possibility of a food additive GTB in PD.

## Introduction

Parkinson’s disease (PD) is the most prevalent movement disorder caused by the demise of dopaminergic neurons present in substantia nigra pars compacta (SNpc) region of the midbrain [1]. Tremor, akinesia and postural instability are the triad symptoms of the patients suffering from this disease [2]. At the pathological level, parkinsonian brains are found to contain intracytoplasmic and neuritic protein inclusion bodies, which are called the Lewy bodies (LB) [3, 4]. The exact etiology of the disease is poorly understood. Therefore, therapeutic interventions to prevent or attenuate dopaminergic cell loss are still failing to reverse the progressiveness of the debilitating disease. Current drugs used to treat patients only offer symptomatic relief, whereas long term intake of these drugs exhibits several unavoidable side effects that compromise the quality of life of patients [5–8].

Fortunately, in the last several decades, experimental research on PD has identified some major causative cellular events that potentially lead to the onset of parkinsonism such as mitochondrial dysfunction [9], oxidative stress [10], inhibited ubiquitin proteasomal system [11], accumulation of *α*-synuclein protein aggregates [12] and inflammation [13]. Over the years, investigations have made it evident that glial cells and glia-induced inflammation have great impact on modulating the demise of nigral dopaminergic neurons [14–18]. *First*, microglial activation is seen in nigra in close proximity to injured or dying neurons [19]. *Second*, the CSF level of nitric oxide and brain level of inducible nitric oxide synthase (iNOS) are higher in PD patients in comparison with a group of patients without dopamine problem [20, 21]. Accordingly, knockdown of iNOS protects dopaminergic neurons in MPTP-intoxicated mice [22]. *Third*, TNF*α*, IL-1*β* and IL-6 are found in CSF or affected brain regions in PD [23]. *Fourth*, transgenic mice that carry homozygous mutation of both the TNF receptors, but not the individual receptors, are resistant to MPTP intoxication [24]. *Fifth*, the transcription of most of the proinflammatory molecules require nuclear factor-*κ*B (NF-*κ*B). It has been found that NF-*κ*B is activated in the SNpc of PD patients and MPTP-intoxicated mice and monkey [14, 15, 25] and that selective inhibition of NF-*κ*B by NEMO-binding domain (NBD) peptides protect dopaminergic neurons in mouse [15] and monkey [25] models of PD. *Sixth*, reports have shown that greater existence of pro-inflammatory M1 microglia over M2 phenotype cause neuronal death in mouse models of PD [26]. Similarly, prevalence of inflammatory A1 astrocytes also imparts harmful effects on the neurons depending on the tissue environment [18]. *Seventh*, certain proinflammatory chemokines such as RANTES and eotaxin produced by activated microglia also participate in the death of dopaminergic neurons [27–29]. Therefore, although glial cells primarily participate in promoting neuronal survival via secretion of neurotrophic factors such as GDNF or BDNF [30–32], excessive and chronic glial activation is detrimental for the survival of dopaminergic neurons and controlling inflammation may be an important step for protecting dopamine in PD and its animal model.

Glyceryl tribenzoate (GTB) or tribenzoin belongs to the family of benzoic acid, which is used as a flavoring ingredient and a food preservative. Our previous studies have established the anti-inflammatory and neuroprotective activity of GTB in mouse models of Huntington’s disease [33] and multiple sclerosis [34]. In the present study, we have evaluated the effect of GTB in treating PD pathology in MPTP-induced mouse and primate models of the disease. While investigating the effect of GTB in mice, we have consistently found the presence of its derivative sodium benzoate (NaB) in the brain and serum of GTB-treated mice. Interestingly, GTB administration protects dopaminergic neurons in SN and striatal dopamine (DA) level in MPTP-intoxicated mice. The protective effect of GTB is also reproduced in MPTP-intoxicated hemiparkinsonian monkeys resulting in attenuated neuronal death and improvement of motor behavior. Protection of dopaminergic neurons by GTB also parallels with narked suppression of inflammatory molecules in SN of hemiparkinsonian monkeys. The results demonstrate the beneficial effect of GTB against PD pathology and provide a hint on using this molecule as a possible therapeutic agent in PD.

## Materials and Methods

### Reagents

GTB was purchased from Spectrum (New Brunswick, NJ). MPTP was procured from Sigma-Aldrich (St. Louis, MO). Mouse anti–TH antibody (ImmunoStar, Hudson, WI), goat anti-Iba1 antibody (Abcam, Cambridge, MA), rabbit anti-GFAP antibody (Agilent Technologies, Santa Clara, CA), mouse anti-iNOS Ab (BD Biosciences, San Jose, CA), anti GTP-p21^Ras^ (New East Biosciences) and anti-phospho-p65 (Abcam) antibodies were purchased from different vendors. Cy2-and Cy5-conjugated secondary antibodies were obtained from Jackson Immuno-Research Laboratories (West Grove, PA).

### MPTP Intoxication In Animals

Mice: C57BL6 mice were purchased from Jackson Laboratories. Animal maintenance was done according to the guidelines of National Institute of Health and was approved by the Institutional Animal Care and Use committee of the Rush University Medical Center (Chicago, IL). During experiments, mice were treated with MPTP intraperitonealy at a dose of 18 mg/kg for four consecutive times, each 2 h apart [14, 15, 17, 29, 30, 32, 35].

Monkey: Female rhesus monkeys (6–8 y old; 5–7 kg) were used in the present study. Each animal was housed in single cage, where the diets were supplemented with fruit during the experiments. The study was performed in accordance with federal guidelines of proper animal care and with the approval of the Institutional Animal Care and Use Committee. During the MPTP intoxication, monkeys were tranquilized with ketamine (10 mg/kg, i.m.) and then maintained on an anaesthetic plane with isoflurane (1–2%). Prior to injection, a right-sided incision was made along the medial edge of the sternocleidomastoid muscle. The carotid sheath was opened for the identification of common carotid artery, internal jugular vein, and vagus nerves. The common carotid artery was exposed below the carotid bifurcation. The external carotid artery was then ligated. A 27-gauge butterfly needle was inserted into the common carotid artery in a direction retrograde to blood flow; for each injection, 20 mL of saline containing 3 mg of MPTP–HCl (Sigma-Aldrich) was infused at a rate of 1.33 mL/min (15 min). After the infusion was completed, 3 mL of saline was delivered and then the incision was closed [25, 36, 37].

### GTB Administration In Animals

Mice: GTB was solubilized in 0.1% methyl cellulose and it remains as a colloidal substance. Mice were treated with GTB at different doses (25, 50 and 100 mg/kg/d) via gavation for 7 days starting from 3 h after MPTP intoxication. Similarly, one group of MPTP-intoxicated mice received only the vehicle, methyl cellulose.

**Figure 1:**
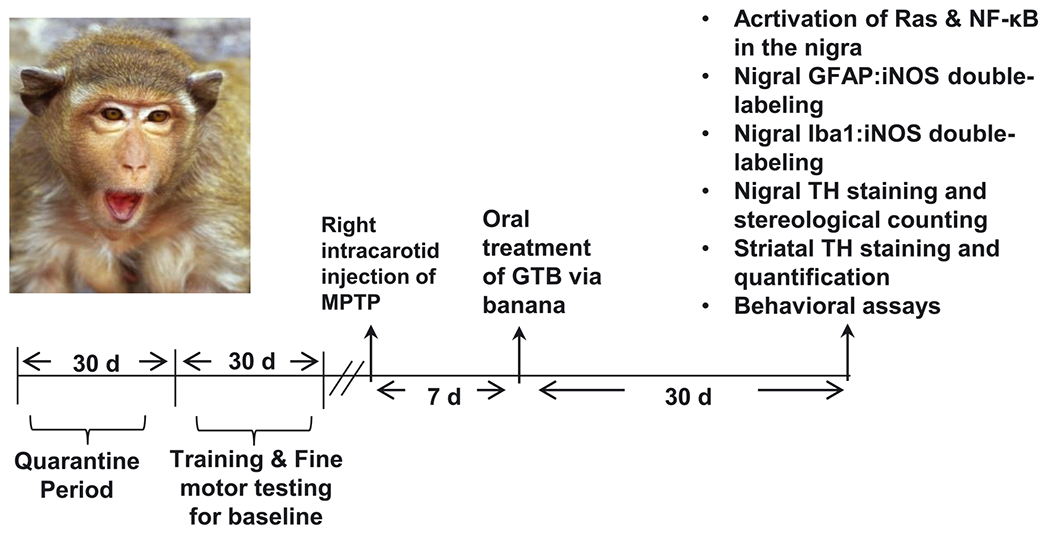
Schematic presentation of experiments in monkeys. Female rhesus monkeys were kept for 30 days in quarantine period and then the animals were subjected to motor training for the next 30 days. Intracarotid injection of MPTP (3 mg/animal) in monkey brain was conducted in the right hemisphere. After 7 days of MPTP intoxication, monkeys showing classical parkinsonian symptoms were fed with GTB via banana daily for the next 30 days. During the 30 d time period, behaviour of animals were monitored thrice in a week. At the end point, monkeys were sacrificed and the brain tissues were collected to perform immunostaining of TH, glial fibrillary acidic protein (GFAP), ionized calcium binding adaptor molecule 1 (Iba1), inducible nitric oxide synthase (iNOS), interleukin-1*β* (IL-1*β*) and GTP-p21^Ras^ in nigral sections. Striatal tissues were collected for HPLC analyses of neurotransmitters.

Monkey: For GTB treatment, only monkeys exhibiting hemiparkinsonian symptoms 7 d after the first intracarotid injection were used. Hemiparkinsonian monkeys were treated with GTB orally at the dose of 50 mg/kg/d through banana for the total of 30 days (Figure 1). Another group of hemiparkinsonian animals received only banana as the vehicle.

### Western Blotting

Mouse midbrain tissues were isolated and homogenized in RIPA buffer 50 mM Tris-HCl, 1 mM EDTA sodium salt, 150 mM NaCl, 1% Nonidet P-40, 0.5% sodium deoxycholate, and protease inhibitor cocktail. The homogenate was centrifuged at 17,500 xg for 10 min and the resulting supernatant was collected for protein estimation using BCA method and further used for Western blotting. Proteins were run in 10% SDS-PAGE and then transferred on to nitrocellulose membrane and probed with anti-TH (1:1000) primary antibodies overnight. Next day corresponding secondary antibody tagged with specific fluorophore (1:10,000; Jackson Immuno-Research) and the blots were scanned under Odyssey infrared scanner (Li-COR, Lincoln, NE). Band intensities were quantified using ImageJ software (NIH, USA) [38–40].

### Immunostaining

Mice were transcardially perfused with 4% paraformaldehyde and brains were kept in 30% sucrose solution. Coronal sections of 40 μm width from the mice brain were taken by cryostat and immunostaining for TH was performed as described earlier [28]. In case of monkeys, animals were anesthetized with pentobarbital (25 mg/kg i.v.) and sacrificed by perfusion with 0.9% saline. Brain was removed from each animal and kept in ice cold saline and further slabbed using monkey brain slicer. Next the tissue slabs were immersed in Zamboni fixative and after that kept in 30% sucrose solution. Sections of 40 μm thickness were taken from the tissue slabs corresponding to midbrain and caudate-putamen region using microtome. The sections were stained for multiple immunostaining using antibodies for TH (1:1000), Iba1 (1:1000), GFAP (1:1000), iNOS (1:500), GTP- p21^Ras^ (1:200), phospho-p65 (1:500) and IL-1*β* (1:200). For immunofluorescence analysis, sections were incubated with Cy2 or Cy5-labelled secondary antibodies, whereas for immunohistochemistry of TH, the staining was developed by using diaminobenzidine (DAB)/H_2_O_2_ (DAB Kit; Vector Laboratories) as described previously [25, 41]. Counting of TH neurons in SN of monkey brains was performed using STEREO INVESTIGATOR software (MicroBrightfield, Williston, VT) having an optical fractionators. Quantification of TH fiber density in the striatum was carried out as described earlier [25, 41].

### HPLC Analysis For Neurotransmitters

Tissues were isolated from multiple regions in the striatum by punching from the tissue slabs. The isolated tissues were processed for HPLC analysis. Briefly, tissues were sonicated in 0.2 M perchloric acid and kept in ice for 30 min. Then the homogenate was centrifuged at 17,500 xg for 10 min at 4 °C and 10 μL of supernatant was injected in an Eicompak SC-3ODS column (Complete Stand-Alone HPLC-ECD System EiCOMHTEC-500; JM Science, Grand Island, NY). The level of dopamine (DA), 3, 4-dihydroxyphenylacetic acid (DOPAC), and homovanillic acid (HVA) was measured as stated earlier [41].

### Behavioral Assays

Experimental monkeys were acclimatized to certain regular performance prior to conducting MPTP intracarotid injection (Figure 1). Following the MPTP intoxication, animal behaviour was rated three times in a week based on parkinsonian scale (PD scale) for clinical symptoms of monkeys by an individual completely blinded to the treatments. The scale consists of ratings on 10 parkinsonian features, which are tremor, posture, locomotion, hypokinesia, bradykinesia, balance, fine and gross motor skills, startle response, and freezing. Delaying feeding time until after the task was completed ensured the animal compliance with the test. Data obtained from the behavioural analysis contained time taken by the animal to reach the fruit placed in the cage (reaction time), time consumed to pick up the fruit while the hand is in the cage (reception time) and the total time taken to move the hand into the chamber, retrieve the fruit, and bring the hand back out of the panel and into the cage (total time) [25, 41].

### HPLC Analysis Of NaB

Mouse brain lysates and monkey serum samples were analyzed for the detection of NaB in Waters 2695 separation module HPLC system with the help of “Em-power pro” software and Phenomenex Luna 5 μm C18 100A column (250 × 4.6 mm; 280 nm UV wavelength). The mobile phase consisted of phase A (acetonitrile with 0.01M H_3_PO_4_) and phase B (H_2_O with 0.01M H_3_PO_4_) (1:4) and the flow rate was kept at 0.5 mL/min. Midbrain tissues were isolated from mice fed with GTB (50 mg/kg) for 7 days and collected in chloroform: methanol (2:1) extraction solvent containing 0.05 M perchloric acid. Aspirin was added as an internal standard. Tissues were homogenized and centrifuged at 20,000 xg for 10 min. Organic and aqueous phases were separated carefully and 10 μL aqueous phase was analyzed for the detection of NaB by HPLC [33]. Similarly, serum samples collected from experimental monkey were processed for chloroform: methanol (2:1) extraction and 10 μL of the aqueous phase was injected into the HPLC column.

### Enzyme Assays

Lactate dehydrogenase (LDH) and serum glutamate–pyruvate transaminase (SGPT) enzyme assays were performed from monkey serum samples using the protocol provided by the manufacturer (Sigma-Aldrich). Standard curve for each assay was prepared separately and the activity of each enzymes in the experimental samples were calculated.

### Statistics

Statistical analyses among multiple samples were performed by one way ANOVA followed by Bonferroni multiple comparison tests. In other cases, where two different groups such as MPTP and MPTP versus MPTP plus GTB were compared, t-test was used to determine statistical significance. For behavioral analysis, one-way repeated measure ANOVA models were used. Values are represented as mean ± SEM.

## Results

### Oral GTB treatment prevents parkinsonian pathology in MPTP-induced mice.

**Figure 2:**
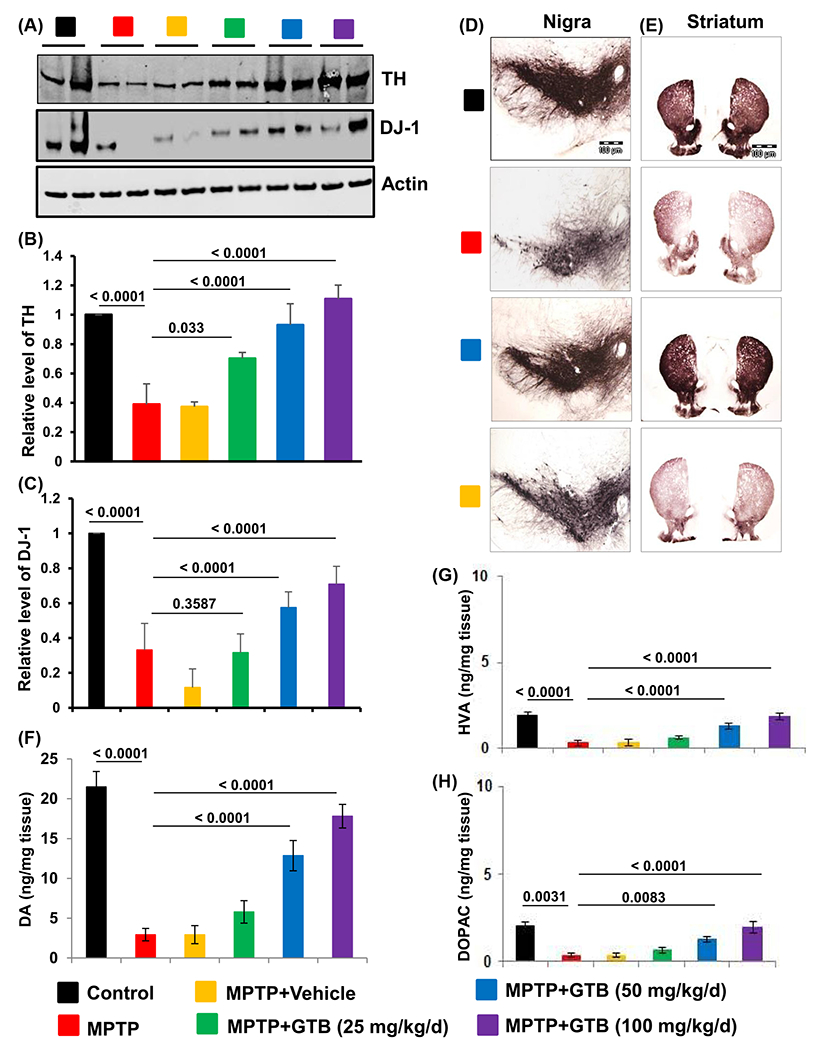
GTB oral administration prevents MPTP-induced parkinsonian pathology in mice. Adult C57BL6 mice (6–8 weeks old) were given acute MPTP intoxication and from the following day mice were administered with different doses of GTB for the next 7 days. Parkinsonian pathologies in these mice were evaluated by monitoring the protein level of tyrosine hydroxylase (TH) and DJ-1 (a, western blot; B, relative level of TH/Actin; C, relative level of dj-1/actin) in nigral tissues, by performing immunohistochemistry for TH in midbrain (D) and striatal (E) sections and by measuring the level of dopamine (DA) (F), 3, 4-dihydroxyphenylacetic acid (DOPAC) (G), and homovanillic acid (HVA) (H) from striatal tissues. One-way ANOVA followed by Bonferroni multiple comparison tests was performed to determine statistical significance. Data are presented as mean ± SEM of five mice per group.

Adult C57BL6 mice were given acute MPTP insults followed by administration with either vehicle or different doses of GTB (25, 50 and 100 mg/kg/d) for the next 7 days. Parkinsonian pathology in these mice was evaluated by monitoring the number of TH positive dopaminergic neurons in SN. The data revealed significant reduction of TH and DJ-1 proteins in SN of MPTP-intoxicated animals (Figure 2A–C). It also accompanies with huge dopaminergic neuronal loss in SN and TH fiber loss at the terminals in striatum (Figure 2D and E). It also accompanies with huge dopaminergic neuronal loss in SN and TH fiber loss at the terminals in striatum (Figure 2C and D). Interestingly GTB administration dose dependently attenuated the MPTP-induced loss of nigral TH level, where optimum protection was found in both 50 mg/kg and 100 mg/kg doses (Figure 2A and B). Next, the level of DA and its metabolites DOPAC and HVA was measured from striatal tissues.

Interestingly GTB administration dose dependently attenuated the MPTP-induced loss of nigral TH and DJ-1 levels, where optimum protection was found in both 50 mg/kg and 100 mg/kg doses (Figure 2A and B). Next, the level of DA and its metabolites DOPAC and HVA was measured from striatal tissues. As expected, almost 80% loss in DA level was found in MPTP-treated striatum. GTB treatment (50 and 100 mg/kg), in parallel to attenuation of nigral TH loss, also markedly protected striatal DA and its metabolites (Figure 2E–G). It signified that 50 mg/kg dose of GTB was sufficient to offer neuroprotection against MPTP-toxicity in mice. Therefore, for further studies, we used GTB at the dose of 50 mg/kg/d. To confirm the protective effect of GTB further in MPTP mouse model, we performed TH immunostaining in mouse brain sections. It demonstrated that 50 mg/kg/d GTB administration significantly protected nigral TH neurons as well as the striatal TH fibers against MPTP-insult and this protection was absent in MPTP-intoxicated animals fed with only vehicle, confirming the effect of GTB in parkinsonism *in vivo* (Figure 2C and D). We also examined whether GTB was metabolized in the body to form its derivative. Therefore, we analysed the midbrain tissue of GTB-fed animals for the presence of GTB metabolite NaB by HPLC analysis. The findings showed clear presence of NaB in the midbrain fraction of GTB-fed animals, but not in the vehicle fed mice (Supplementary Figure 1A–C).

### Oral administration of GTB inhibits MPTP-induced activation of p21^Ras^ and NF-*κ*B in the nigra of hemiparkinsonian monkeys.

**Figure 3:**
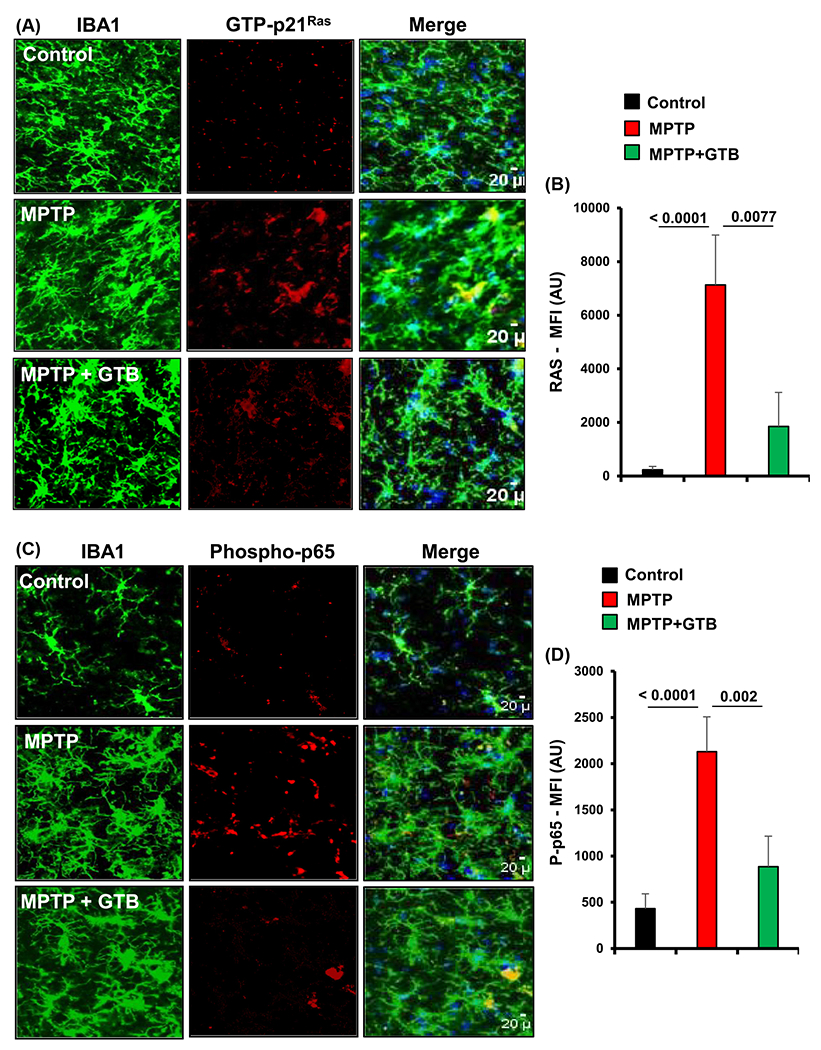
GTB treatment prevents the activation of p21^Ras^ and NF-*κ*B *in vivo* in the nigra of hemiparkinsonian monkeys. Naïve female rhesus monkeys received a right intracarotid injection of MPTP. After 7 d of injection, monkeys displaying classical parkinsonian postures received GTB (50 mg/kg body wt/d) orally via banana. After 30 d of treatment, nigral sections were double-labelled for Iba1 and GTP-p21^Ras^ (A) and Iba1 with phospho-p65 (C). Mean fluorescence intensity (MFI) analyses of GTP-p21^Ras^ (B) and phospho-p65 (D) were performed using ImageJ. At least two different sections from each brain were considered for the MFI analyses. One-way ANOVA followed by Bonferroni multiple comparison tests was performed to determine statistical significance. **p<0.01 and ***p<0.001 indicate significance compared to respective groups. Data are presented as mean ± SEM of four monkeys per group.

After establishing the neuroprotective dose of GTB in mice, we carried out our experiments in MPTP-induced hemiparkinsonian monkey, which closely mimic the pathological features of PD. In this experiment, only monkeys displaying parkinsonian symptoms 7 d after the first intracarotid injection were administered with GTB (50 mg/kg/d) orally for the next 30 days (Figure 1). First, we performed immunofluorescence analyses from the nigral sections to evaluate the effect of GTB on inflammation. As previously demonstrated by Ghosh and co-workers that the metabolite of MPTP, MPP^+^, activates microglial p21^Ras^ to stimulate the NF-*κ*B mediated up-regulation of inflammatory molecules in glial cells [14], we primarily monitored the status of GTP-p21^Ras^ (activated Ras) and the activated form of NF-*κ*B (phospho-p65), which is required for nuclear localization and transcriptional up-regulation of inflammatory genes, in Iba1 positive microglia of SN. Results exhibited remarkably higher presence of GTP-p21^Ras^ (Figure 3A and B) and phospho-p65 (Figure 3C and D) in microglia present in the ipsilateral (injected) side of the brain. In contrast, level of these two proteins involved in the inflammatory cascade was significantly down-regulated in GTB-fed hemiparkinsonian monkeys. The immunostaining showed that following GTB treatment, expression level of microglial GTP-p21^Ras^ and phospho-p65 became almost similar to that found in the contralateral (control) side of the brain. Overall, the data indicate that GTB is able to inhibit induction of inflammatory signalling pathway triggered by MPTP intoxication.

### Oral administration of GTB inhibits the induction of inflammatory molecules in the nigra of hemiparkinsonian monkeys.

**Figure 4:**
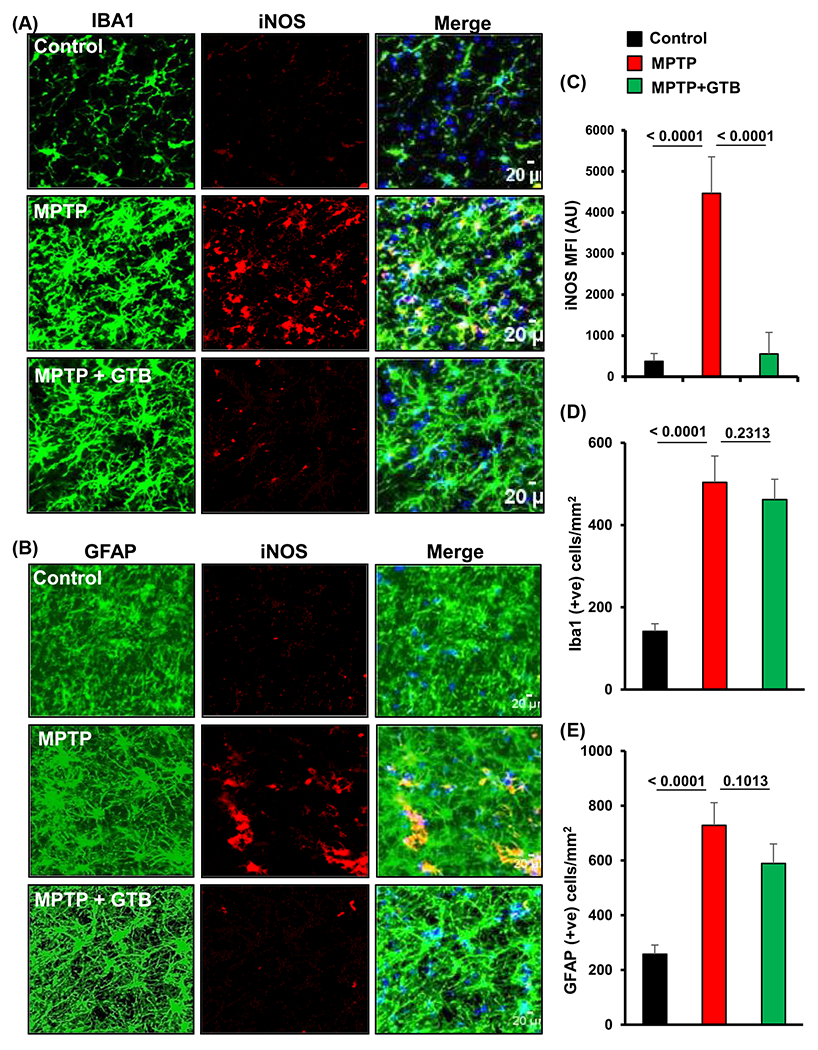
Oral GTB administration inhibits expression of iNOS in glial cells in the nigra of hemiparkinsonian monkeys. Monkeys were unilaterally injected with MPTP and following 7 days of MPTP intoxication, monkeys were fed with GTB (50 mg/kg/d) via banana. Expression of the inflammatory marker iNOS was evaluated in Iba1 positive microglia. (A) and GFAP positive astrocytes (B) in nigra of monkey brains. The MFI of iNOS in nigral sections were measured by ImageJ (C). Statistical analyses were conducted by one-way ANOVA followed by Bonferroni multiple comparison tests. **p<0.01 and ***p<0.001 indicate significance compared to respective groups. Values are shown as mean ± SEM of four monkeys per group.

Inhibition in the activation of p65 in microglia by GTB prompted us to monitor the expression of downstream inflammatory molecules expressed in microglia. For that purpose, double immunofluorescence staining was performed for inflammatory marker, inducible nitric oxide synthase (iNOS) in Iba1 positive microglia and GFAP positive astrocytes. The results showed significant up-regulation of iNOS expression in both microglia and astrocytes in SN of MPTP-treated monkey brain (Figure 4A–C). Interestingly, as a consequence to the inhibition of p65 activation, GTB treatment also suppressed the level of iNOS in midbrain. Similarly, induction of the major inflammatory cytokine, IL-1*β* level was also investigated by immunostaining in microglia. Parallel to the inhibition of iNOS expression in microglia, GTB also inhibited MPTP-induced IL-1*β* expression in microglia present in SN of monkeys (Supplementary Figure 2A and B). Interestingly the number of microglia and astrocytes was not found to be decreased by GTB when compared to the only MPTP-treated group (Figure 4A and B). It suggests that GTB specifically inhibits glial inflammation without decreasing the number of microglia and astrocytes in the brain.

### Oral GTB treatment protects nigral dopaminergic neurons and striatal TH fibers in MPTP-intoxicated monkeys.

**Figure 5:**
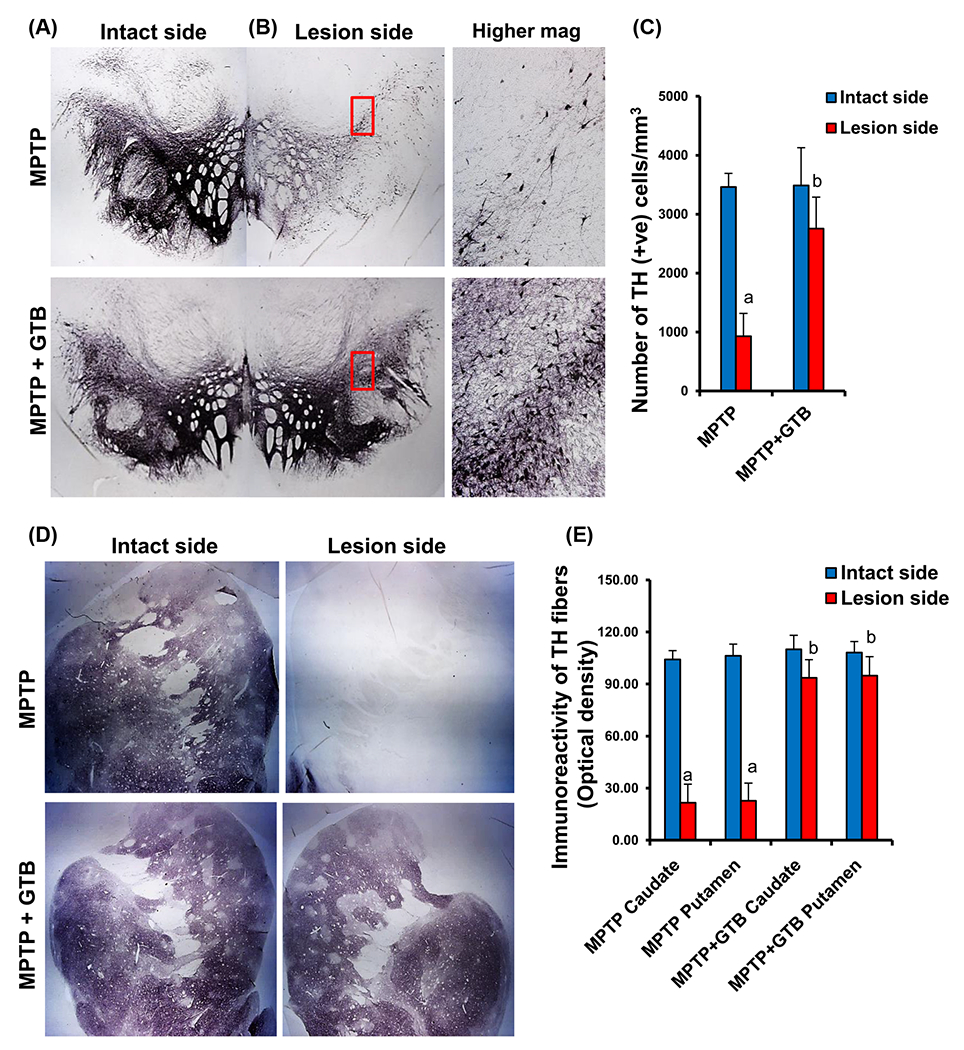
GTB treatment protects the nigrostriatum in hemiparkinsonian monkeys. Naïve female rhesus monkeys received a right intracarotid injection of MPTP. After 7 d of injection, monkeys displaying classical parkinsonian postures received GTB (50 mg/kg body wt/d) orally via banana. After 30 d of treatment, nigral sections were immunostained for TH ((A), lower magnification; (B), higher magnification). Estimates of dopaminergic nigral cell number were performed bilaterally using an unbiased design-based counting method (optical fractionator, Stereo investigator; Microbrightfield, williston, VT) (C). Counting of TH neurons was performed by a single investigator blinded to the experimental conditions. Striatal sections were immunostained for TH (D). Density of TH fibers in caudate and putamen (E) is expressed as optical density. Analysis of six different striatal sections of each of four monkeys (n=4) was performed by a single investigator blinded to the experimental conditions. ^a^*p*<0.001 vs MPTP-intact; ^b^*p*<0.001 vs MPTP-lesion. Statistical analyses were conducted by one-way ANOVA followed by Bonferroni multiple comparison tests.

**Figure 6:**
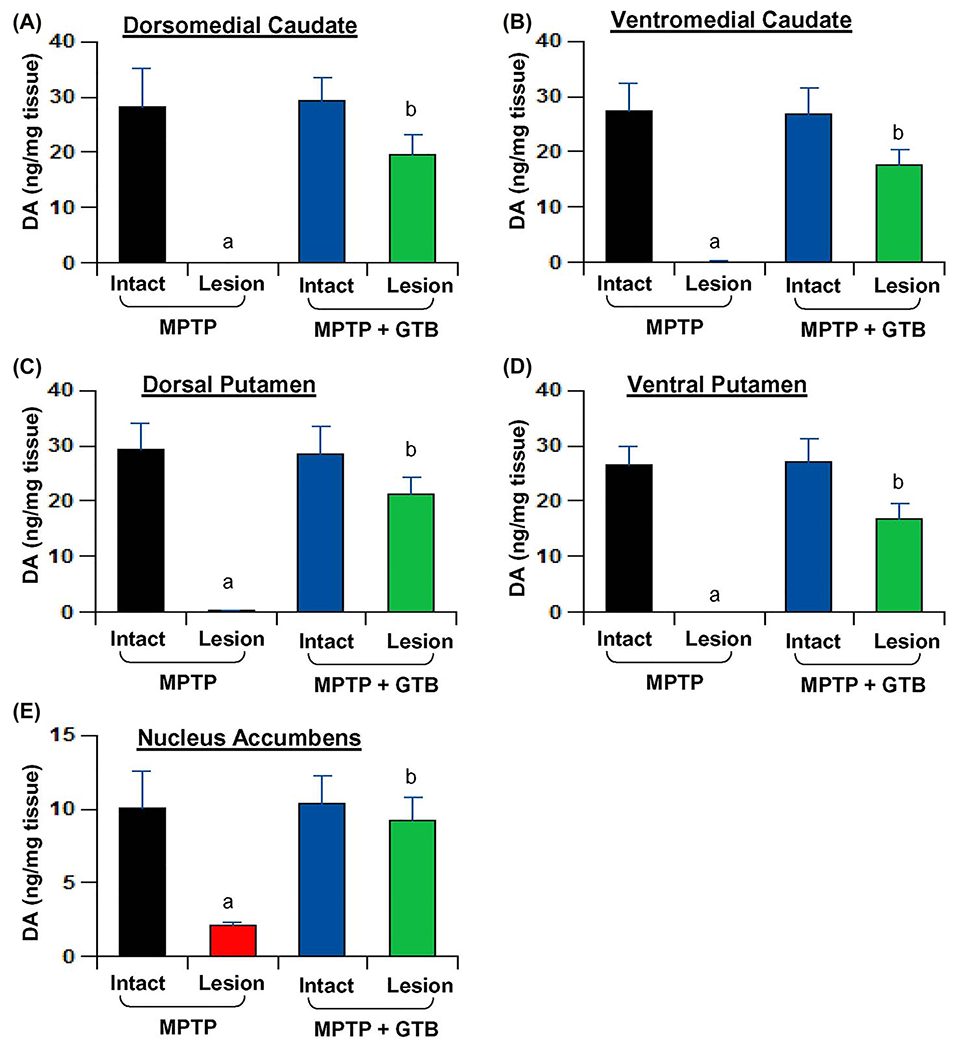
Oral GTB protects striatal DA in hemiparkinsonian monkeys. Naïve female rhesus monkeys received a right intracarotid injection of MPTP. After 7 d of injection, monkeys displaying classical parkinsonian postures received GTB (50 mg/kg body wt/d) orally mixed with banana. MPTP group of monkeys also received normal banana as control. After 30 d of treatment, DA levels were measured in dorsomedial caudate. (A), ventromedial caudate (B), dorsal putamen (C), ventral putamen (D), and nucleus accumbens (E). HPLC quantification of DA was performed by a single investigator blinded to the experimental conditions. Data are presented as mean ± SEM of four monkeys per group (n=4). ^a^*p*<0.001 vs MPTP-intact; ^b^*p*<0.001 vs MPTP-lesion.

In the next phase of the study, we assessed the effect of GTB against MPTP-induced parkinsonian pathology by looking at the number of viable TH neurons in SN and its fibers in caudate-putamen region of the basal ganglia. The TH immunostaining convincingly showed severe loss of nigral TH neurons in the lesioned side of the brain, where the number of viable neurons was almost 70% less than that of the intact side (Figure 5A–C). Nigral TH loss, as expected, resulted in huge loss of TH fibers in caudate and putamen (Figure 5D and E). However, by GTB administration both nigral neurons and the nerve terminals in caudate-putamen were significantly restored and it was almost comparable to the intact side of the brain as reflected from the TH cell body counting in SN (Figure 5C) and TH fiber optical density in striatum (Figure 5E). It suggests that reduction in glial inflammation by GTB positively correlates with attenuation of TH neuronal loss in the nigrostriatal axis. To address the functional outcome of restoration of dopaminergic neurons in brain, we also measured the level of DA and its metabolites in multiple regions of basal ganglia and also in nucleus accumbens. We found consistent depletion of DA in dorsomedial (Figure 6A) and ventromedial caudate (Figure 6B) as well as in dorsal and ventral putamen (Figure 6C and D). In addition, there was loss of DA in nucleus accumbens (Figure 6E) and that must be the result of death of certain dopaminergic population in ventral tegmental area of the midbrain. GTB, along with the restoration of TH terminals in striatum, prevented the loss of DA in all the forebrain areas examined in the study.

### Oral GTB treatment results in behavioural improvement in hemiparkinsonian monkeys.

**Figure 7:**
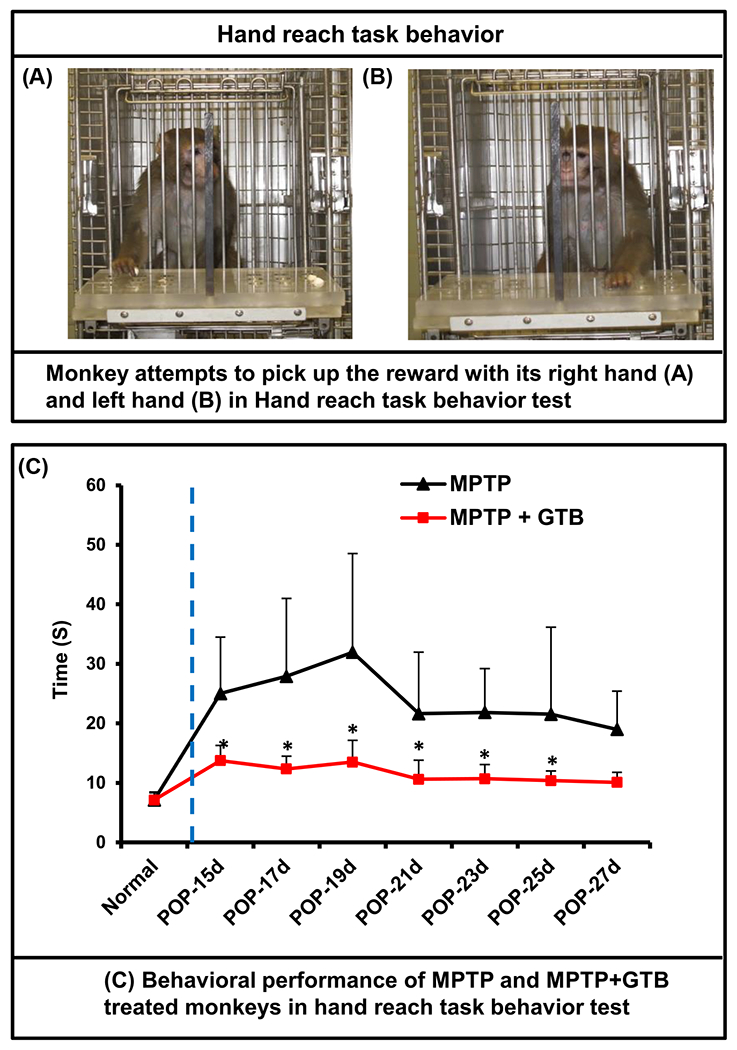
Oral GTB treatment improved the performance of hemiparkinsonian monkeys on hand-reach task. Naïve female rhesus monkeys received a right intracarotid injection of MPTP. After 7 d of injection, monkeys displaying classical parkinsonian postures received GTB (50 mg/kg body wt/d) orally via banana. MPTP group of monkeys also received normal banana as control. On different days of treatment, monkeys were examined for the hand-reach task. Picture of monkeys attempting to pick up the reward with its right hand (A) and left hand (B) in hand-reach task behavior. Time taken by monkeys during the hand-reach task using the left hand was monitored and presented in the diagram (C). Statistical significance was measured repeated measure ANOVA. Results are mean ± SEM of four monkeys per group (n=4). *p<0.05 vs MPTP.

Remarkable protection of nigral dopaminergic neurons and striatal DA level by GTB in hemiparkinsonian monkeys made it indispensable for us to examine the behavioural or phenotypical improvement of MPTP-induced monkeys. As stated earlier, behaviour of experimental monkeys including hand-reaching task, general activity and clinical dysfunction (tremor, posture, locomotion, bradykinesia, balance, fine and gross motor skills, startle response, and freezing) were rated thrice in a week based on the PD scale. Similar to the earlier findings [41], MPTP caused reduced movement of the left arm (contralateral to the injected side) of monkeys, whereas expectedly the movement of the right arm was much faster as evidenced by the time taken by the left arm in performing the tasks during the experiment (Figure 7). Importantly, oral GTB treatment significantly improved the motor ability as revealed from the subjective clinical rating scale and objective operant motor test (Table 1). Most importantly, monkeys receiving oral treatment of GTB via banana did not show any untoward signs such as, vomiting, diarrhoea, psychosis, hyperkinesia, hair loss, etc., suggesting that oral GTB intake might be devoid of side effects.

### GTB reduces cytotoxicity markers in serum of hemiparkinsonian monkeys.

**Figure 8:**
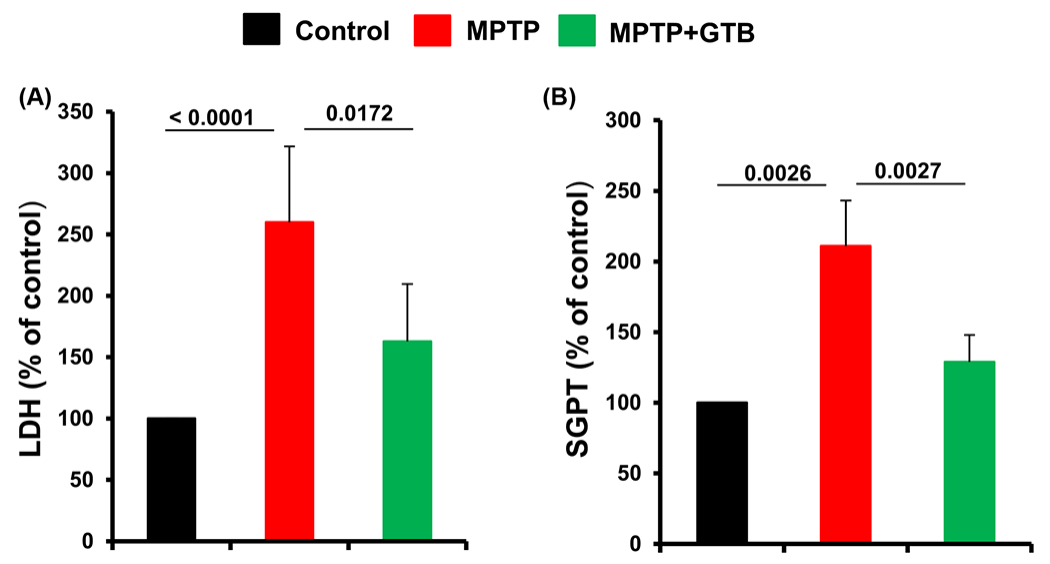
Oral GTB treatment reduces cytotoxicity in hemiparkinsonian monkeys. Level of cytotoxicity in MPTP and MPTP plus GTB-treated monkeys were assessed by monitoring the activity of lactate dehydrogenase (LDH) and serum alanine aminotransferase (ALT) in serum of animals. Values are represented as percentage compared to the untreated control animals. Statistical analyses were conducted by one way ANOVA followed by Bonferroni multiple comparison tests. **p<0.01 and ***p<0.001 indicate significance compared to respective groups. Values are shown as mean ± SEM of four monkeys per group.

Lastly, in the present study, we examined the level of certain cytotoxicity markers in serum of untreated and GTB-treated monkeys. Higher serum LDH than normal levels usually indicates tissue damage. Similarly, alanine aminotransferase (ALT) is probably the most widely used clinical biomarker of liver well-being. Accordingly, we observed marked upregulation of both LDH and ALT in the serum of hemiparkinsonian monkeys. Interestingly, along with the correction of parkinsonian pathologies by GTB oral administration, serum LDH and ALT levels were also down-regulated (Figure 8A and B). It clearly suggests attenuation of cell death by GTB in MPTP-intoxicated monkeys.

## Discussion

The debilitating effects of gliosis and inflammation on dopaminergic neurodegeneration have been well manifested by multiple studies in toxin-induced models [15, 42], in *α*-synuclein injected animals [43, 44] and also in human PD brains [45]. The current understanding about the disease pathology states that glial activation and inflammation is a consequence of neuronal death. However, exaggerated microglial activation and generation of pro-inflammatory cytokines such as IL-1*β*, TNF-*α*, IL-6 are also shown to be prerequisite for the demise of nigral dopaminergic neurons in different models of PD [46–48]. Moreover, brain inflammation coupled with the interference of adaptive immune cells is known to be associated with progressiveness of the disease [27, 29]. Therefore, molecules inhibiting inflammatory cascades and the resultant neuroinflammation provide promising effects in delaying PD progression [49, 50] and that is also evidenced by the fact that people taking NSAIDs for long term poses less risk from PD [51, 52].

The significance of the present study is oral administration of a simple food additive, GTB, prevents MPTP-induced neuronal death not only in mice but also in primate model of PD. The MPTP-induced mice model recapitulates major parkinsonian pathologies including nigral dopaminergic cell death, exaggerated inflammation and striatal dopamine loss [15, 53]. Therefore, the study was initiated in mice with the aim to evaluate the anti-parkinsonian effect of GTB and to find out the optimum dose of the molecule. However, there are certain caveats associated with the acute MPTP-mice model as the progressive mode of neuronal demise and persistent inflammation are not found in mice brains. Perhaps that is one of the reasons for studies, conducted in mice, not efficiently translated in humans. Therefore, MPTP-induced monkey model was chosen to further strengthen the anti-parkinsonian effect of GTB. Rhesus monkeys are old world monkeys, which share the common ancestor, a primitive anthropoid, with the humans. Almost 93% of the DNA sequence of Rhesus monkeys matches with that of the humans [54]. More importantly, the immune system of monkeys is very much similar to the human immune system. The parkinsonian features observed in MPTP-intoxicated monkeys are progressive and irreversible and therefore this model is the best available alternative to study the anti-parkinsonian effect of GTB oral administration.

The key points of the present investigation are, first GTB dose-dependently increased nigral TH level and the neuroprotective protein DJ-1 expression in MPTP-intoxicated mice, where 50 mg/kg dose exhibited the optimum neuroprotection. As TH and DJ-1 up-regulation is considered as the reversal of MPTP-induced parkinsonian effects obtained by GTB oral treatment [55], this finding sets the ground of further studies on its anti-parkinsonian effect in a more relevant model. Second, the level of GTP-p21^Ras^ and phospho-p65, which play roles upstream of the induction of inflammatory molecules, are significantly inhibited by GTB oral administration in MPTP-induced monkeys. Our previous report showed that the toxic metabolite of MPTP, MPP^+^, activates GTP-p21^Ras^, which further leads to activation of NF-*κ*B as evidenced by the increased level of phospho-p65 in microglia and astrocytes in SN of MPTP-intoxicated mice [14]. Suppression of GTP-p21^Ras^ and phospho-p65 in microglia of GTB-fed animals clearly suggests that induction of inflammatory cascade is inhibited by GTB. Third, as the downstream effect of NF-*κ*B inhibition, the expression of inflammatory marker proteins IL-1*β* and iNOS was also remarkably reduced in glial cells in GTB-fed animals proving the anti-inflammatory effect of this molecule in PD. It also indicates that down-regulation of IL-1*β* and iNOS provides better environment for neuronal survival in SN because both these inflammatory markers are known to cause neuronal death in PD [56–58]. Importantly, the effect of GTB in preventing inflammation was specific as we did not observe any reduction in the number of glial cells in SN. Fourth, suppression of inflammation in SN happens in parallel to attenuation of nigral dopaminergic cell death and striatal TH fibers in GTB-fed animals. Supporting the notion that prevention of inflammation protects dopaminergic neurons in PD, our study convincingly demonstrated that following GTB treatment MPTP-induced neuronal death is markedly halted in SN resulting in greater abundance of the major neurotransmitter DA in caudate and putamen of the experimental monkeys. Fifth, neuroprotection exerted at the pathological level by GTB is also reflected in the motor behavior of the animals as these monkeys retained the motor skills, which are seriously compromised in only MPTP-injected animals. Lastly, GTB administration reduces the overall toxicity and cells death associated with MPTP-intoxication in animals as evidenced by the reduced level of serum LDH and ALT in GTB-fed group. Along with these important findings, our study also revealed that availability of NaB, a GTB metabolite and a FDA-approved drug used for the treatment for urea cycle disorders, increased in the body of both MPTP-intoxicated mice and monkeys. Our previous reports have clearly shown that NaB prevents glial inflammation and up-regulates DJ-1 and parkin level in nigral tissues [55] and also promotes alleviation *α*-synuclein down-regulation in the progressive A53T model of Lewy body dementia [59]. Moreover, NaB treatment was shown to augment secretion of neurotrophic GDNF from the astrocytes and prevent MPTP-induced parkinsonian features in mice [32]. Therefore, it might be speculated that the neuroprotective effect exerted by GTB oral administration might also partly be caused by NaB synthesis in the body.

In summary, we have demonstrated that oral administration of GTB, an FDA-approved food additive, decreases the activation glial cells in the midbrain, protects nigral dopaminergic neurons, restores striatal dopamine, and improves the behavioral functions in MPTP-intoxicated hemiparkinsonian monkeys. Since the monkey model is the best available model for PD, our results highlight an important neuroprotective effect of GTB and suggest that GTB may be repurposed for therapeutic interference in PD.

## Supplementary Material

Supplemental figures

## Figures and Tables

**Table 1:** Oral GTB treatment led to functional improvements in hemiparkinsonian monkeys.

Motor parameters	Before MPTP infusion	After MPTP infusion	After treatment	
			MPTP + banana	MPTP + GTB-banana
General (gross) motor skills (0-3)				
R Hand	0.00 ± 0.00	0.12 ± 0.01	0.07 ± 0.04	0.01 ± 0.00
L Hand	0.00 ± 0.00	1.93 ± 0.12	1.17 ± 0.88	0.71 ± 0.03
Tremor (0-3)				
R Hand	0.00 ± 0.00	0.21 ± 0.03	0.00 ± 0.00	0.00 ± 0.00
L Hand	0.00 ± 0.00	1.24 ± 0.32	1.07 ± 0.70	0.17 ± 0.01^a^
Balance (0-3)	0.00 ± 0.00	1.75 ± 0.28	1.19 ± 0.50	0.42 ± 0.04^a^
Posture (0-3)	0.00 ± 0.00	1.50 ± 0.43	1.35 ± 0.50	0.28 ± 0.03^a^
Bradykinesia (0-5)	0.00 ± 0.00	2.12 ± 0.32	1.81 ± 0.42	0.71 ± 0.11^b^
Gait (0-5)	0.00 ± 0.00	1.75 ± 0.05	1.63 ± 0.83	0.36 ± 0.05^a^
Defense reaction (0-2)	0.00 ± 0.00	1.28 ± 0.15	1.5 ± 0.08	0.23 ± 0.07^a^
Freezing (0-2)	0.00 ± 0.00	0.92 ± 0.14	0.81 ± 0.23	0.29 ± 0.02^a^
Total (sum mean)	0.00 ± 0.00	12.82 ± 1.85	10.6 ± 4.18	3.18 ± 0.36^a^

GTB treatment led to functional improvements in hemiparkinsonian monkeys. Naïve female rhesus monkeys received a right intracarotid injection of MPTP. After 7 d of injection, monkeys displaying classical parkinsonian postures received GTB (50 mg/kg body wt/d) orally via banana. Monkeys (n=4 in each group) were rated thrice a week using a parkinsonian rating scale (PD scale) utilized to quantify the clinical status of the monkeys. The scale included ratings of 10 parkinsonian features (tremor, posture, locomotion, hypokinesia, bradykinesia, balance, fine and gross motor skills, startle response, and freezing). Monkeys were not tested on these tasks during the week between MPTP intoxication and initiation of GTB treatment.

b *p*<0.01 &

a *p*<0.001 vs MPTP + banana.

## References

[1] Pattan V, Seth S, Jehangir W, Bhargava B, Maulik SK. Effect of atorvastatin and pioglitazone on plasma levels of adhesion molecules in non-diabetic patients with hypertension or stable Angina or both. J Clin Med Res 2015;7:613–9. 26124907 10.14740/jocmr2178e PMC4471748

[2] Vila M, Przedborski S. Genetic clues to the pathogenesis of Parkinson’s disease. Nat Med 2004;10:S58–62. 15272270 10.1038/nm1068

[3] Spillantini MG, Crowther RA, Jakes R, Hasegawa M, Goedert M. alpha-Synuclein in filamentous inclusions of Lewy bodies from Parkinson’s disease and dementia with lewy bodies. Proc Natl Acad Sci U S A 1998;95:6469–73. 9600990 10.1073/pnas.95.11.6469 PMC27806

[4] Wakabayashi K, Tanji K, Mori F, Takahashi H. The Lewy body in Parkinson’s disease: molecules implicated in the formation and degradation of alpha-synuclein aggregates. Neuropathology 2007;27:494–506. 18018486 10.1111/j.1440-1789.2007.00803.x

[5] Dutta D, Mohanakumar KP. Tea and Parkinson’s disease: constituents of tea synergize with antiparkinsonian drugs to provide better therapeutic benefits. Neurochem Int 2015;89:181–90. 26271432 10.1016/j.neuint.2015.08.005

[6] Moller JC, Oertel WH. Pramipexole in the treatment of advanced Parkinson’s disease. Eur J Neurol 2000;7:21–5. 11054155 10.1046/j.1468-1331.2000.0070s1021.x

[7] Tintner R, Jankovic J. Treatment options for Parkinson’s disease. Curr Opin Neurol 2002;15:467–76. 12151845 10.1097/00019052-200208000-00011

[8] Youdim MB. The path from anti Parkinson drug selegiline and rasagiline to multifunctional neuroprotective anti Alzheimer drugs ladostigil and m30. Curr Alzheimer Res 2006;3:541–50. 17168653 10.2174/156720506779025288

[9] Beal MF. Mitochondria, oxidative damage, and inflammation in Parkinson’s disease. Ann N Y Acad Sci 2003;991:120–31. 12846981 10.1111/j.1749-6632.2003.tb07470.x

[10] Sherer TB, Betarbet R, Greenamyre JT. Pathogenesis of Parkinson’s disease. Curr Opin Investig Drugs 2001;2:657–62. 11569943

[11] Betarbet R, Sherer TB, Greenamyre JT. Ubiquitin-proteasome system and Parkinson’s diseases. Exp Neurol 2005;191:S17–27. 15629758 10.1016/j.expneurol.2004.08.021

[12] Brundin P, Ma J, Kordower JH. How strong is the evidence that Parkinson’s disease is a prion disorder? Curr Opin Neurol 2016;29:459–66. 27257944 10.1097/WCO.0000000000000349 PMC5054685

[13] Troncoso-Escudero P, Parra A, Nassif M, Vidal RL. Outside in: unraveling the role of neuroinflammation in the progression of Parkinson’s disease. Front Neurol 2018;9:860. 30459700 10.3389/fneur.2018.00860 PMC6232883

[14] Ghosh A, Roy A, Matras J, Brahmachari S, Gendelman HE, Pahan K. Simvastatin inhibits the activation of p21ras and prevents the loss of dopaminergic neurons in a mouse model of Parkinson’s disease. J Neurosci 2009;29:13543–56. 19864567 10.1523/JNEUROSCI.4144-09.2009 PMC2862566

[15] Ghosh A, Roy A, Liu X, Kordower JH, Mufson EJ, Hartley DM, Selective inhibition of NF-kappaB activation prevents dopaminergic neuronal loss in a mouse model of Parkinson’s disease. Proc Natl Acad Sci U S A 2007;104:18754–9. 18000063 10.1073/pnas.0704908104 PMC2141849

[16] Pahan P, Pahan K. Can cinnamon bring aroma in Parkinson’s disease treatment? Neural Regen Res 2015;10:30–2. 25788911 10.4103/1673-5374.150647 PMC4357107

[17] Roy A, Ghosh A, Jana A, Liu X, Brahmachari S, Gendelman HE, Sodium phenylbutyrate controls neuroinflammatory and antioxidant activities and protects dopaminergic neurons in mouse models of Parkinson’s disease. PLoS One 2012;7:e38113. 22723850 10.1371/journal.pone.0038113 PMC3377667

[18] Yun SP, Kam T-I, Panicker N, Kim SM, Oh Y, Park J-S, Block of A1 astrocyte conversion by microglia is neuroprotective in models of Parkinson’s disease. Nat Med 2018;24:931–8. 29892066 10.1038/s41591-018-0051-5 PMC6039259

[19] Wu DC, Jackson-Lewis V, Vila M, Tieu K, Teismann P, Vadseth C, Blockade of microglial activation is neuroprotective in the 1-methyl-4-phenyl-1, 2, 3, 6-tetrahydropyridine mouse model of Parkinson disease. J Neurosci 2002;22:1763–71. 11880505 10.1523/JNEUROSCI.22-05-01763.2002 PMC6758858

[20] Hunot S, Boissiere F, Faucheux B, Brugg B, Mouatt-Prigent A, Agid Y, Nitric oxide synthase and neuronal vulnerability in Parkinson’s disease. Neuroscience 1996;72:355–63. 8737406 10.1016/0306-4522(95)00578-1

[21] Qureshi GA, Baig S, Bednar I, Sodersten P, Forsberg G, Siden A. Increased cerebrospinal fluid concentration of nitrite in Parkinson’s disease. Neuroreport 1995;6:1642–4. 8527732 10.1097/00001756-199508000-00013

[22] Dehmer T, Lindenau J, Haid S, Dichgans J, Schulz JB. Deficiency of inducible nitric oxide synthase protects against MPTP toxicity in vivo. J Neurochem 2000;74:2213–6. 10800968 10.1046/j.1471-4159.2000.0742213.x

[23] Bessler H, Djaldetti R, Salman H, Bergman M, Djaldetti M. IL-1 beta, IL-2, IL-6 and TNF-alpha production by peripheral blood mononuclear cells from patients with Parkinson’s disease. Biomed Pharmacother 1999;53:141–5. 10349502 10.1016/S0753-3322(99)80079-1

[24] Sriram K, Matheson JM, Benkovic SA, Miller DB, Luster MI, O’Callaghan JP. Deficiency of TNF receptors suppresses microglial activation and alters the susceptibility of brain regions to MPTP-induced neurotoxicity: role of TNF-alpha. FASEB J 2006;20:670–82. 16581975 10.1096/fj.05-5106com

[25] Mondal S, Roy A, Jana A, Ghosh S, Kordower JH, Pahan K. Testing NF-kappaB-based therapy in hemiparkinsonian monkeys. J Neuroimmune Pharmacol 2012;7:544–56. 22661311 10.1007/s11481-012-9377-9 PMC3419771

[26] Moehle MS, West AB. M1 and M2 immune activation in Parkinson’s Disease: foe and ally? Neuroscience 2015;302:59–73. 25463515 10.1016/j.neuroscience.2014.11.018 PMC4442748

[27] Chandra G, Roy A, Rangasamy SB, Pahan K. Induction of adaptive immunity leads to nigrostriatal disease progression in MPTP mouse model of Parkinson’s disease. J Immunol 2017;198:4312–26. 28446566 10.4049/jimmunol.1700149 PMC5467696

[28] Chandra G, Rangasamy SB, Roy A, Kordower JH, Pahan K. Neutralization of RANTES and eotaxin prevents the loss of dopaminergic neurons in a mouse model of Parkinson disease. J Biol Chem 2016;291:15267–81. 27226559 10.1074/jbc.M116.714824 PMC4946939

[29] Dutta D, Kundu M, Mondal S, Roy A, Ruehl S, Hall DA, RANTES-induced invasion of Th17 cells into substantia nigra potentiates dopaminergic cell loss in MPTP mouse model of Parkinson’s disease. Neurobiol Dis 2019;132:104575. 31445159 10.1016/j.nbd.2019.104575 PMC6834904

[30] Gottschalk CG, Jana M, Roy A, Patel DR, Pahan K. Gemfibrozil protects dopaminergic neurons in a mouse model of Parkinson’s disease via PPARalpha-dependent astrocytic GDNF pathway. J Neurosci 2021;41:2287–300. 33514677 10.1523/JNEUROSCI.3018-19.2021 PMC8018777

[31] Jana A, Modi KK, Roy A, Anderson JA, van Breemen RB, Pahan K. Up-regulation of neurotrophic factors by cinnamon and its metabolite sodium benzoate: therapeutic implications for neurodegenerative disorders. J Neuroimmune Pharmacol 2013;8:739–55. 23475543 10.1007/s11481-013-9447-7 PMC3663914

[32] Patel D, Jana A, Roy A, Pahan K. Cinnamon and its metabolite protect the nigrostriatum in a mouse model of Parkinson’s disease via astrocytic GDNF. J Neuroimmune Pharmacol 2019;14:503–18. 31119595 10.1007/s11481-019-09855-0 PMC6708487

[33] Dutta D, Majumder M, Paidi RK, Pahan K. Alleviation of Huntington pathology in mice by oral administration of food additive glyceryl tribenzoate. Neurobiol Dis 2021;153:105318. 33636386 10.1016/j.nbd.2021.105318 PMC8026693

[34] Mondal S, Dasarathi S, Pahan K. Glyceryl tribenzoate: a flavoring ingredient, inhibits the adoptive transfer of experimental allergic encephalomyelitis via TGF-beta: implications for multiple sclerosis therapy. J Clin Cell Immunol 2017;8:488. 28367355 10.4172/2155-9899.1000488 PMC5373804

[35] Khasnavis S, Roy A, Ghosh S, Watson R, Pahan K. Protection of dopaminergic neurons in a mouse model of Parkinson’s disease by a physically-modified saline containing charge-stabilized nanobubbles. J Neuroimmune Pharmacol 2014;9:218–32. 24122363 10.1007/s11481-013-9503-3

[36] Kordower JH, Emborg ME, Bloch J, Ma SY, Chu Y, Leventhal L, Neurodegeneration prevented by lentiviral vector delivery of GDNF in primate models of Parkinson’s disease. Science 2000;290:767–73. 11052933 10.1126/science.290.5492.767

[37] Roy A, Mondal S, Kordower JH, Pahan K. Attenuation of microglial RANTES by NEMO-binding domain peptide inhibits the infiltration of CD8(+) T cells in the nigra of hemiparkinsonian monkey. Neuroscience 2015;302:36–46. 25783477 10.1016/j.neuroscience.2015.03.011 PMC4527882

[38] Dutta D, Jana M, Majumder M, Mondal S, Roy A, Pahan K. Selective targeting of the TLR2/MyD88/NF-kappaB pathway reduces alpha-synuclein spreading in vitro and in vivo. Nat Commun 2021;12:5382. 34508096 10.1038/s41467-021-25767-1 PMC8433339

[39] Dutta D, Ali N, Banerjee E, Singh R, Naskar A, Paidi RK, Low levels of prohibitin in substantia nigra makes dopaminergic neurons vulnerable in Parkinson’s disease. Mol Neurobiol 2018;55:804–21. 28062948 10.1007/s12035-016-0328-y

[40] Raha S, Ghosh A, Dutta D, Patel DR, Pahan K. Activation of PPARalpha enhances astroglial uptake and degradation of beta-amyloid. Sci Signal 2021;14:eabg4747. 34699252 10.1126/scisignal.abg4747 PMC8793854

[41] Mondal S, Rangasamy SB, Roy A, Dasarathy S, Kordower JH, Pahan K. Low-dose maraviroc, an antiretroviral drug, attenuates the infiltration of T cells into the central nervous system and protects the nigrostriatum in hemiparkinsonian monkeys. J Immunol 2019;202:3412–22. 10.4049/jimmunol.1800587 PMC6824976 31043478

[42] Lee E, Hwang I, Park S, Hong S, Hwang B, Cho Y, MPTP-driven NLRP3 inflammasome activation in microglia plays a central role in dopaminergic neurodegeneration. Cell Death Differ 2019;26:213–28. 29786072 10.1038/s41418-018-0124-5 PMC6329843

[43] Harms AS, Delic V, Thome AD, Bryant N, Liu Z, Chandra S, alpha-Synuclein fibrils recruit peripheral immune cells in the rat brain prior to neurodegeneration. Acta Neuropathol Commun 2017;5:85. 29162163 10.1186/s40478-017-0494-9 PMC5698965

[44] Lastres-Becker I, Ulusoy A, Innamorato NG, Sahin G, Rabano A, Kirik D, alpha-Synuclein expression and Nrf2 deficiency cooperate to aggravate protein aggregation, neuronal death and inflammation in early-stage Parkinson’s disease. Hum Mol Genet 2012;21:3173–92. 22513881 10.1093/hmg/dds143

[45] Croisier E, Moran LB, Dexter DT, Pearce RK, Graeber MB. Microglial inflammation in the parkinsonian substantia nigra: relationship to alpha-synuclein deposition. J Neuroinflammation 2005;2:14. 15935098 10.1186/1742-2094-2-14 PMC1177985

[46] Emmer KL, Waxman EA, Covy JP, Giasson BI. E46K human alpha-synuclein transgenic mice develop Lewy-like and tau pathology associated with age-dependent, detrimental motor impairment. J Biol Chem 2011;286:35104–18. 21846727 10.1074/jbc.M111.247965 PMC3186371

[47] Tofaris GK, Garcia Reitbock P, Humby T, Lambourne SL, O’Connell M, Ghetti B, Pathological changes in dopaminergic nerve cells of the substantia nigra and olfactory bulb in mice transgenic for truncated human alpha-synuclein(1-120): implications for Lewy body disorders. J Neurosci 2006;26:3942–50. 16611810 10.1523/JNEUROSCI.4965-05.2006 PMC6673887

[48] Watson MB, Richter F, Lee SK, Gabby L, Wu J, Masliah E, Regionally-specific microglial activation in young mice over-expressing human wildtype alpha-synuclein. Exp Neurol 2012;237:318–34. 22750327 10.1016/j.expneurol.2012.06.025 PMC3443323

[49] Han X, Sun S, Sun Y, Song Q, Zhu J, Song N, Small molecule-driven NLRP3 inflammation inhibition via interplay between ubiquitination and autophagy: implications for Parkinson disease. Autophagy 2019;15:1860–81. 30966861 10.1080/15548627.2019.1596481 PMC6844502

[50] Hirsch EC, Hunot S. Neuroinflammation in Parkinson’s disease: a target for neuroprotection? Lancet Neurol 2009;8:382–97. 19296921 10.1016/S1474-4422(09)70062-6

[51] Gagne JJ, Power MC. Anti-inflammatory drugs and risk of Parkinson disease: a meta-analysis. Neurology 2010;74:995–1002. 20308684 10.1212/WNL.0b013e3181d5a4a3 PMC2848103

[52] Gao X, Chen H, Schwarzschild MA, Ascherio A. Use of ibuprofen and risk of Parkinson disease. Neurology 2011;76:863–9. 21368281 10.1212/WNL.0b013e31820f2d79 PMC3059148

[53] Muralikrishnan D, Mohanakumar KP. Neuroprotection by bromocriptine against 1-methyl-4-phenyl-1, 2, 3, 6-tetrahydropyridine-induced neurotoxicity in mice. FASEB J 1998;12:905–12. 9657530 10.1096/fasebj.12.10.905

[54] Gibbs RA, Rogers J, Katze MG, Bumgarner R, Weinstock GM, Mardis ER, Evolutionary and biomedical insights from the rhesus macaque genome. Science 2007;316:222–34. 17431167 10.1126/science.1139247

[55] Khasnavis S, Pahan K. Sodium benzoate, a metabolite of cinnamon and a food additive, upregulates neuroprotective Parkinson disease protein DJ-1 in astrocytes and neurons. J Neuroimmune Pharmacol 2012;7:424–35. 21701815 10.1007/s11481-011-9286-3 PMC3189510

[56] Ferrari CC, Pott Godoy MC, Tarelli R, Chertoff M, Depino AM, Pitossi FJ. Progressive neurodegeneration and motor disabilities induced by chronic expression of IL-1beta in the substantia nigra. Neurobiol Dis 2006;24:183–93. 16901708 10.1016/j.nbd.2006.06.013

[57] Pott Godoy MC, Tarelli R, Ferrari CC, Sarchi MI, Pitossi FJ. Central and systemic IL-1 exacerbates neurodegeneration and motor symptoms in a model of Parkinson’s disease. Brain 2008;131:1880–94. 18504291 10.1093/brain/awn101 PMC2442423

[58] Tapias V, Hu X, Luk KC, Sanders LH, Lee VM, Greenamyre JT. Synthetic alpha-synuclein fibrils cause mitochondrial impairment and selective dopamine neurodegeneration in part via iNOS-mediated nitric oxide production. Cell Mol Life Sci 2017;74:2851–74. 28534083 10.1007/s00018-017-2541-x PMC5524146

[59] Raha S, Dutta D, Roy A, Pahan K. Reduction of lewy body pathology by oral cinnamon. J Neuroimmune Pharmacol 2020. 10.1007/s11481-020-09955-2. PMC7933354 32889602

