# Supplementary material for: Protection of dopaminergic neurons in hemiparkinsonian monkeys by flavouring ingredient glyceryl tribenzoate
Source: NeuroImmune Pharm Ther. 2022 Jun 8;1(1):7–22. doi: 10.1515/nipt-2022-0005 (PMC9212717; doi:10.1515/nipt-2022-0005)
Supplement: Supplementary file 1 [file j_nipt-2022-0005_suppl.pdf]

Protection of dopaminergic neurons in hemiparkinsonian monkeys by flavouring ingredient glyceryl tribenzoate

Suresh B. Rangasamy[1,*], Debashis Dutta[1,*], Susanta Mondal[1,*], Moumita Majumder[1], Sridevi Dasarathy[1], Goutam Chandra[1,&], and Kalipada Pahan[1,2]

[1]Department of Neurological Sciences, Rush University Medical Center, Chicago, USA; [2]Division of Research and Development, Jesse Brown Veterans Affairs Medical Center, Chicago, USA

*First three authors have equal contribution to the work.

[&]Current address: Center for Development and Aging Research, Inter-University Center for Biomedical Research & Super Specialty Hospital, Mahatma Gandhi University Campus at Thalappady, Kottayam 686009 Kerala, India

Running title: Glyceryl tribenzoate for PD

Corresponding author:
Kalipada Pahan, Ph.D.
Department of Neurological Sciences
Rush University Medical Center
1735 West Harrison St
Suite Cohn 310
Chicago, IL 60612
Tel: (312) 563-3592; Fax: (312) 563-3571
Email: Kalipada_Pahan@rush.edu

Number of figures: 8

Number of tables: 1

Number of pages: 23

Acknowledgements: This study was supported by grants (NS83054, NS108025 and AT10980) from NIH to KP. Moreover, KP is the recipient of a Research Career Scientist Award (1IK6 BX004982) from the Department of Veterans Affairs.

Supplementary Figure 1. Detection of sodium benzoate (NaB) in midbrain of GTB fed animals. Adult C57BL6 mice were administered with either vehicle (0.1% methyl cellulose) or with 50 mg/kg/d GTB by gavation for 7 days and midbrain tissues were isolated and processed for LC-MS. Aspirin was used as internal control. Standard of aspirin (10 ng) and NaB (10 ng) was run in the HPLC to obtain the retention times for each of the molecules (A). Then aqueous fractions (10 µl) isolated from brain of vehicle-treated (B) and GTB-treated (C) animals were injected into the column to detect NaB. Fractions isolated from at least three animal brains were used for the experiment.

Supplementary Figure 2. Oral GTB administration inhibits expression of IL-1β in microglia present in the nigra of hemiparkinsonian monkeys. Monkeys were unilaterally injected with MPTP and following 7 days of MPTP intoxication, monkeys were treated with GTB (50 mg/kg/d) via banana. Expression of the inflammatory marker IL-1β in microglia was evaluated by double-immunostaining of Iba1 and IL-1β (A). The MFI of IL-1β was measured using ImageJ (B). Two different section per brain were used for the staining and analysis. Statistical analysis was performed by one-way ANOVA followed by Bonferroni multiple comparison tests. *$p< 0.05$, **$p< 0.01$ and ***$p< 0.001$ indicate significance compared to respective groups. Values are shown as mean ± SEM of four monkeys per group.

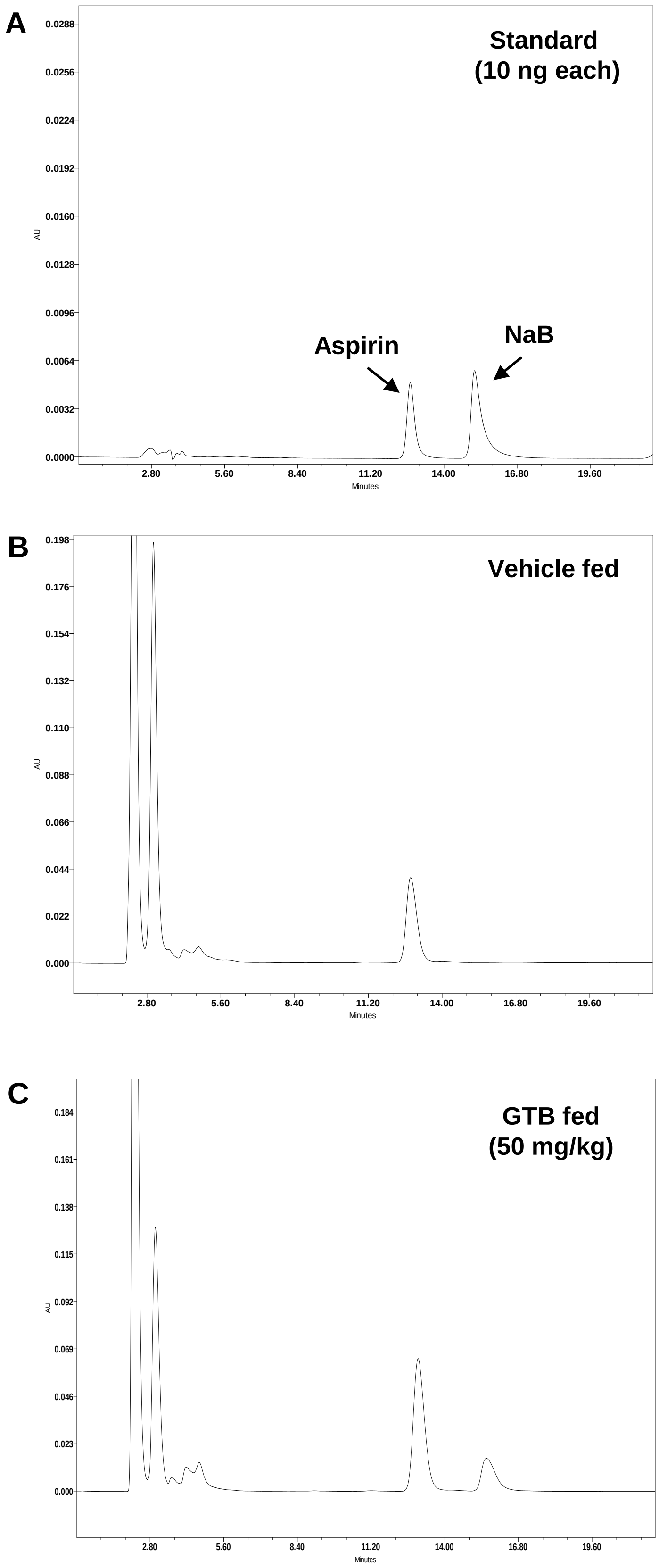

Supplementary Fig 1

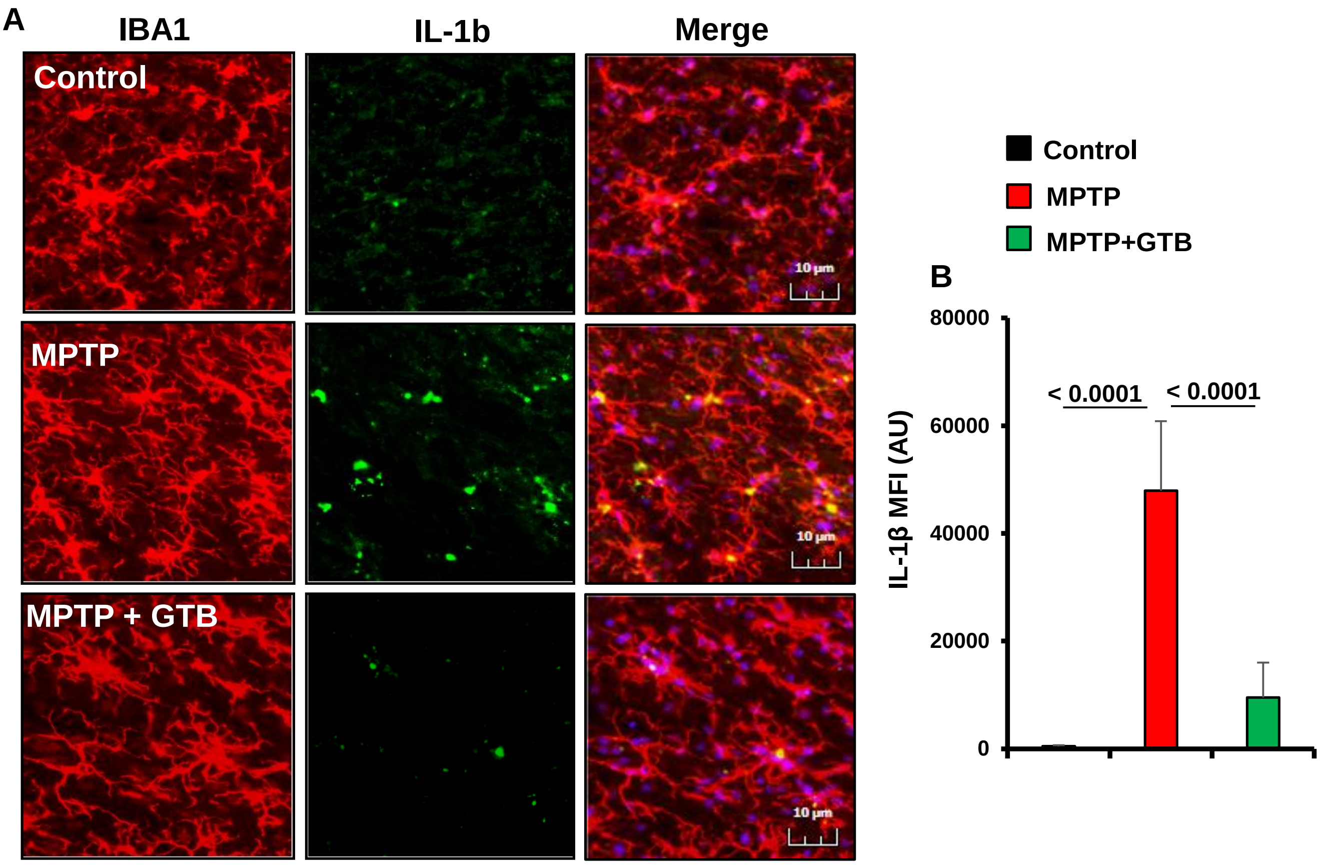

Supplementary Fig. 2
